# Labeling and intergenerational transmission of crime: The interaction between criminal justice intervention and a convicted parent

**DOI:** 10.1371/journal.pone.0172419

**Published:** 2017-03-08

**Authors:** Sytske Besemer, David P. Farrington, Catrien C. J. H. Bijleveld

**Affiliations:** 1 Institute of Human Development, University of California, Berkeley, California, United States of America; 2 Institute of Criminology University of Cambridge, Cambridge, United Kingdom; 3 Netherlands Institute for the Study of Crime and Law Enforcement, VU University Amsterdam, Amsterdam, The Netherlands; Swinburne University of Technology, AUSTRALIA

## Abstract

Labeling theory suggests that criminal justice interventions amplify offending behavior. Theories of intergenerational transmission suggest why children of convicted parents have a higher risk of offending. This paper combines these two perspectives and investigates whether labeling effects might be stronger for children of convicted parents. We first investigated labeling effects within the individual: we examined the impact of a conviction between ages 19–26 on self-reported offending behavior between 27–32 while controlling for self-reported behavior between 15–18. Our results show that a conviction predicted someone’s later self-reported offending behavior, even when previous offending behavior was taken into account. Second, we investigated whether having a convicted parent influenced this association. When we added this interaction to the analysis, a labeling effect was only visible among people with convicted parents. This supports the idea of cumulative disadvantage: Labeling seems stronger for people who are already in a disadvantaged situation having a convicted parent.

## Introduction

Labeling theory predicts that criminal justice interventions amplify offending behavior [1–4]. Similarly, theories of intergenerational transmission predict that children of convicted parents might have a higher risk of offending [5–10]. This paper combines these two perspectives and investigates whether labeling effects might be stronger for children of convicted parents compared with children whose parents have not been convicted. Our paper argues that labeling effects are particularly strong for individuals with convicted parents and almost absent for those without convicted parents.

Investigating this combination of intergenerational transmission and labeling will increase knowledge on the origins and development of criminal behavior, which is vital in preventing intergenerational transmission of criminal behavior. This knowledge is particularly relevant for governments, because labeling implies that government (policies) might be partly responsible for sustaining (intergenerational transmission of) criminal behavior. These issues are especially salient in this time where we see a tendency to sentence offenders increasingly harshly, with a growing reliance on (long) imprisonment (see e.g. [11–15]).

Criminal or antisocial parents appear to be the strongest family factor predicting offending, but it is still unclear why this happens [6]. Farrington [6] has described several mechanisms that could explain this intergenerational transmission, one of which is official bias. As described in detail previously [16], this mechanism hypothesizes that official justice systems, such as the police and the court, are biased against known criminal families. As a result, they pay more attention to these families, which means that family members are more likely to be caught and thus appear in official statistics more often. This explanation asserts that there is not necessarily a real transmission of behavior; there only seems to be an association because children of convicted parents will be caught more frequently than children without convicted parents. Besemer, Farrington, & Bijleveld [16] demonstrated support for this mechanism of official bias.

An important concept related to official bias in intergenerational transmission is labeling. Labeling theory suggests that criminal justice interventions amplify offending behavior. To clarify, labeling occurs when someone’s offending behavior increases after involvement in the criminal justice system. Official bias is defined in an intergenerational context; children of convicted parents have a higher risk of conviction because official justice systems pay more attention to these children; these children’s self-reported offending may or may not be higher than the behavior of children of unconvicted parents. The current paper extends the findings of Besemer et al. [16] by combining these two perspectives and investigating whether labeling effects might be stronger for children of convicted parents compared with children whose parents have not been convicted.

### Labeling theory

Labeling theory suggests that people’s behavior is influenced by the label attached to them by society [1–4]. This label can be a critical factor to a more persistent criminal life course for individuals who might just be experimenting with delinquent activity. Previous studies have shown a considerable impact of convictions on subsequent criminal behavior [17–25]. Revised versions of labeling theory distinguish two major theoretical perspectives of how labeling works [26–28].

First, being labeled might increase an individual’s association with delinquent individuals and influence his or her self-perceptions, attitudes, and beliefs [1,2,21,27,29–31]. As a result of conforming to the criminal stereotype, these individuals will amplify their offending behavior. Also, people might identify more with deviant social groups after receiving a criminal label [29].

Second, people might be pushed into a criminal lifestyle as a result of the potential blockage of conventional and non-criminal pathways. Raphael [32] describes several challenges faced by former inmates who try to find stable jobs, including stigma against ex-offenders by potential employers, less extensive work histories, or behaviors unsuitable for workplaces outside prison, developed while incarcerated. Moreover, a conviction might have a negative impact on educational attainment, which in turn might increase offending, as revealed in the Rochester Youth Development Study [17,33].

Additionally, some people might be more susceptible to labeling effects than others, depending on offenders’ characteristics and on the type of criminal justice sentence received. For example, labeling might have a stronger effect with younger offenders, for whom personality and behavior are presumably more malleable [17,29], and Cullen and Jonson [34] hypothesize that labeling is stronger when sanctions are punitively oriented.

Related to this is the concept of cumulative disadvantage where labeling effects are stronger for those who are already socially and economically disadvantaged [21,35–38]. Foster and Hagan [36] describe how labeling excludes children of convicted parents from society, emphasizing the specific accumulation process for children of convicted parents throughout the life course. This also connects to research showing how the current culture of mass incarceration seems to generate social inequalities [39–41].

A theory related to labeling theory, is Sherman’s defiance theory [42–44]. Sherman stresses the importance of emotions and legitimacy for effectiveness of a sentence. This is based on reintegrative shaming theory [45], proposing that punishment should be aimed to “shame the act, but not the actor” [44]. When a sentence creates a feeling that offenders are being excluded from the society that punished them, they may develop pride that results in an increase and/or persistence of their offending. According to Sherman, defiance occurs when four conditions are present: (1) the offender perceives a punishment as unfair, (2) the offender feels alienated or is poorly bonded to the person or sanctioning agency, (3) the offender perceives the sanction as stigmatizing and targeted at his person instead of at his law-breaking act, and (4) the offender does not acknowledge the shame that the punishment caused him to suffer. Farrington [18] and Murray, Blokland, Farrington, & Theobald [24] indeed found an increase in hostility towards the police after a conviction. Below we first discuss previous research on the combination of labeling and intergenerational transmission of criminal behavior.

### Previous research on labeling and intergenerational transmission

Hagan and Palloni [46] were the first to link these two processes with their paper on the reproduction of a social class. Using data from the *Cambridge Study in Delinquent Development*, they investigated the impact of a conviction (son’s labeling) and a parental conviction (parents’ labeling: official bias). They found support for the idea of the ‘social reproduction of a criminal class’, a process in which the criminal justice system is responsible for the reproduction of criminal behavior of offenders’ children through their treatment of these children [46]. They demonstrated that labeling effects were stronger for people with a convicted father compared with people whose fathers had not been convicted. Unfortunately, their design suffered from methodological flaws. They treated several measures of self-reported offending as independent, when this was not actually the case. For example, they treated self-reported offending at ages 16–17 as independent of self-reported offending at ages 14–15 and used self-reported offending at ages 16–17 to predict self-reported offending at ages 18–19. This is problematic, because self-reported offending at ages 16–17 is measured up to that age and therefore includes offences at ages 14–15. Similarly, self-reported offending at ages 16–17 overlaps with self-reported offending at ages 18–19 (which referred to the previous three years) and therefore it is not possible to treat them as independent variables. Because of these flaws, it is important to replicate this study using independent measures to investigate whether the effect found by Hagan and Palloni [46] is valid. We extend the previous research by Hagan and Palloni [46] and by doing so, we add to the emerging literature of the impact of the social context in labeling processes by focusing on the social context of the family and investigating the cumulative effect of labeling when one has a convicted parent.

Moreover, most studies examining labeling effects have investigated this only up to the age of 22, whereas this study will look at offending behavior until age 32. Most studies have focused on contact with the criminal justice system during the teenage years, and few studies followed respondents to age where adult roles should be established [21]. An exception is the study by Murray et al. [24], who demonstrated robust relationships between juvenile conviction and adult criminal behavior, antisocial personality and multiple life outcomes such as employment, relationships, and mental health up to age 48. It is important to look at offending behavior into adulthood since offending after the early twenties might indicate a more serious offending pattern. Deviant behavior peaks in adolescence [47,48] and it is quite common to display some antisocial behavior during this period. It is, however, a sign of greater deviance if such behavior continues after adolescence or starts in adulthood. It is vital to examine how labeling impacts offending in the long run. The current paper improves upon the narrow focus on short-term effects of official intervention often found in previous research on labeling by investigating labeling effects up to age 32.

More importantly, when studying labeling effects, it is crucial to observe the temporal sequence of the labeling event and subsequent deviant behavior, while controlling for differences in deviant behavior before the labeling event occurred. The majority of previous studies investigating labeling effects [17–19,29,49,50] have failed to clearly distinguish these periods. Kaplan and Johnson [51] and Johnson et al. [52] separated these periods clearly by investigating delinquency at time 1, justice system involvement at time 2 and delinquency at time 3. Murray et al. [24] also separate these periods well. However, Bernburg and Krohn [17], for example, measured official intervention at ages 13.5–16.5 while controlling for self-reported offending at ages 14–16. Another example is West and Farrington [18,25] who compared people with and without a conviction between ages 14–18 on their self-reported offending between these same ages (while controlling for self- reported offending before the age of 14). West & Farrington [18,25] attempted to more clearly separate these periods by examining a small subset of people who were first convicted after age sixteen. They show some evidence of worsening behavior after a conviction; their self-reported offending only started to deteriorate after age sixteen and not between fourteen and sixteen. By not separating these periods in time, it is unknown whether the self-reported offending behavior measured has not already increased because of a conviction during this period. It is crucial to know people’s self-reported offending behavior before they were first convicted and compare the level of self-reported offending after the conviction. This study improves on previous research into labeling by clearly separating these periods in time.

### Current study

We will first investigate whether an offspring conviction increases individuals’ offending behavior. Next, the interaction between someone’s own conviction and a convicted parent will be investigated. Using data from the *Cambridge Study in Delinquent Development* (CSDD) the following hypotheses will be studied:

1. A conviction subsequently increases the number of an individual’s self-reported offences: there is a significant relationship between having a conviction between ages 19 and 26 and self-reported offending between ages 27 and 32, after control- ling for the level of self-reported offending between ages 15 and 18.
2. This labeling effect is stronger for people whose parents have been convicted.

## Method

### Participants

The *Cambridge Study in Delinquent Development* (CSDD) is a prospective longitudinal study that has followed 411 London males born in 1953–54. At the time they were first contacted in 1961–1962, these males were all living in a working-class inner-city area of South London. The sample was chosen by taking all of the boys who were then aged 8–9 and on the registers of six state primary schools within a one-mile radius of a research office that had been established. Hence, the most common year of birth for these males was 1953. In nearly all cases (94 percent), their family bread- winner in 1961–1962 (usually the father) had a working class occupation (skilled, semi-skilled, or unskilled manual worker). Most of the boys were white and of British origin. Donald J. West originally directed the study and David P. Farrington, who has worked on it since 1969, has directed it since 1982. The males have been studied at frequent intervals between the ages of eight and fifty. Information about convictions and self-reported delinquency was collected over the course of these years. Additionally, police records of the parents of these 411 males have been collected. For more information and major findings see West [53], West and Farrington [25,54], Farrington and West [55], Farrington [56,57], Farrington et al. [58,59], Piquero, Farrington, and Blumstein [48], and Farrington, Piquero, and Jennings [60].

The *CSDD* has many strengths that make it unique to investigate labeling effects [46]: a) the study is based on a community sample; b) the study has a prospective longitudinal design; c) the study started before the onset of official offending when they boys were age eight; d) it includes repeated measures of both self-reported crime and criminal records; e) the study has a long follow-up period with high retention rates (93% at age 48); f) an extremely rich range of data were collected on the participants and their families from childhood.

### Materials and procedure

#### Self-reported offending

Self-reported offending was measured at ages 18 and 32 and referred to the periods between ages 15–18 and 27–32. At age 18, 389 (95%) of the original males were interviewed, and 378 (94%) at age 32. Males who did not have an interview at both ages were excluded from the current analyses. Eighty-nine per cent of 411 men were interviewed at both ages, which resulted in a sample of 365 males. See [58] for more information on data collection of the self-reported data. The self-report offenses were presented on cards, and the males were initially asked to sort the cards according to whether or not they had committed each act during a specified reference period. Where the men had reading difficulties, the cards were read out to them. More detailed questions were then asked about the offences reported, such as how many times the person had done it, the age he had first done it, and the age he had last done it. The exact wording of the items at the different ages are shown in [61]. Ten types of offences were enquired about: burglary, theft of motor vehicles, theft from motor vehicles, shoplifting, theft from machines, theft from work, fraud, assault, drug use and vandalism. For the current analyses, a sum of the total number of self-reported offences was used. Drug use and fraud were not included in the sum variable, since drug use had a different scale and distribution up to 1,000 (while the others had a scale up to 100) and previous analyses showed that the ratio between self-reported and official convictions for drug use and fraud is high: the chances of being caught for these offences are low [58]. If drug offences had been included, they would disproportionately dominate the sum variable for self-reported offending.

#### Official convictions

Official offending of both parents and offspring was measured using official criminal records. Convictions were searched in the Criminal Record Office in London [62]. The date when the offence was committed was used to time the delinquency. If no commission date was known, the conviction date was used. Offences were defined as acts leading to convictions, and only one offence per day was counted. This rule was adopted so that each separate behavioral act could yield only one offence; if all offences had been counted, the number of offences would have been greater than the number of criminal behavioral acts, resulting in an overestimation of criminal behavioral acts [58]. Convictions were counted for relatively serious offending ranging from theft, burglary, fraud to robbery, sexual offences and murder. Minor offences such as drunkenness and traffic offences were excluded.

#### Outcome variable

Self-reported offending was measured between ages 15–18 and 27–32. For both hypothesis 1 and 2, labeling effects were examined and thus the level of self-reported offending was measured between ages 27 and 32.

#### Predictor variables

The independent variables for hypotheses 1 and 2 were whether people had been convicted during time 2 (19–26 years) and their level of self-reported offending for time 1 (15–18 years). For hypothesis 2, the variable parental conviction until the offspring’s 15th birthday was added to the analysis.

#### Control variables

We included several three sets of control variables in the analyses: impulsive behavior by the son, socioeconomic status of the family and parenting variables. The CSDD has collected three dichotomized variables related to the son’s impulsive behavior:

- teacher rating on “lacks concentration/restless in class” measured at ages 8 and 10.
- mother/peer rating on “daring/takes many risks in climbing, traffic, exploring etc.” (mother at age 8 and peer at age 10).
- psychomotor clumsiness/impulsivity on three psychomotor tests at ages 8 and 10: Porteus maze, spiral maze, tapping test.

For a more detailed description and earlier use of these variables see [63] and [64]. The three variables were correlated. Therefore, these risk factors were summarized by taking their mean value (if one variable was missing, the mean of the remaining variables was automatically calculated). This resulted in a combined impulsivity variable reflecting a son’s impulsive and risk taking behavior in childhood.

The CSDD has several dichotomous risk factor variables that measure low socio-economic status of the parent when the boy was aged eight to ten: low occupational prestige, low family income, poor housing, large family, (low) education of father, and (low) education of mother. Low occupational prestige indicated that the family breadwinner (usually the father) had an unskilled manual job. Low family income and poor housing were rated by the study social workers who interviewed the families; poor housing indicated dilapidated premises [58]. Similar to the impulsivity variables, the six SES variables correlated with each other and were summarized by taking the mean value. Similar to the combined impulsivity variable, if one variable was missing, the mean of the remaining variables was automatically calculated.

Finally, we included a variable indicating poor child rearing, which was a combination of harsh-erratic discipline and parental conflict, rated by psychiatric social workers based on interviews with parents at age 8.

### Analytic approach

First, we examined whether there was a significant relationship between a conviction between ages 19 and 26 (time 2) and the level of self-reported offending between ages 27 and 32 (time 3), while controlling for the level of self-reported offending between ages 15 and 18 (time 1). We chose to control for the self-reported offending during time 1, since the self-reported offending during time 2 might have been impacted already by a conviction during that period. Negative binomial regression was used because the dependent variable (self-reported offending between ages 27 and 32) was highly skewed. With such a skewed distribution it was inappropriate to run a linear regression analysis. Negative binomial regression analysis suitably deals with skewed distributions. Furthermore, the predictor variable (self-reported offending between ages 15 and 18) was similarly skewed and therefore log-transformed in the analysis.

Second, to investigate whether the impact of a conviction was stronger for people whose parents have been convicted, the interaction between the variables of having a conviction between ages 19–26 and having a convicted parent was investigated. An interaction term (conviction 19–26 * parental conviction) was added to the negative binomial regression. The predictor variables were centered around the mean before analyzing them in the regression analysis. Centering variables around the mean is recommended when investigating interaction effects in multiple regression analysis [65].

Third, we investigated whether the seriousness of offspring offending impacted the relationship between labeling and having a convicted parent. To examine this, the sum of self-reported burglary and violence measured at age 18 was used (self-reported offending measured at age 32 was the outcome variable). This seriousness variable was added to the regression analysis to test the interaction between a parental conviction and offspring conviction on offspring self-reported offending. Furthermore, we examined whether the seriousness of parents’ convictions impacted on this relationship. A dichotomous variable was used that was coded 1 when parents had been convicted for burglary, robbery, assault, wounding, insulting or threatening behavior, sexual offences, murder, manslaughter, drug or weapon offences.

Fourth, we adjusted for a son’s impulsive and/or risk taking behavior and socioeconomic status by adding these two variables to the regression analysis, first separately and then in one big model including all predictors.

## Results

### Labeling: The impact of a conviction on subsequent offending

Two hundred and seventy individuals did not have a conviction before their 19th birthday. Thirty-one of these were convicted between their 19th and 27th birthday and these were compared with the 239 people who had not been convicted in either of these periods. The results in Table 1 (model 1) demonstrate that having a conviction between the 19th and 27th birthday (time 2) and the level of self-reported offending between the 15th and 19th birthday (time 1) were both significant predictors of the level of self-reported offending between the 27th and 32*nd* birthday (time 3). A conviction predicted someone’s later self-reported offending behavior, even when previous offending behavior was taken into account. These results support the idea of labeling.

**Table 1:** The impact of a conviction between ages 19–26 (time 2) for offspring with no previous convictions on level of self-reported offending between ages 27–32 (time 3) while controlling for the level of self- reported offending between ages 15–18 (time 1) and the interaction with parental conviction (up to offspring’s 15^th^ birthday).

Dependent variable: Self-reported offending ages 27–32	Model 1				Model 2			
	B	95% CI B		*p*	B	95% CI B		*p*
Convicted 19–26 or not	0.90	0.51-	1.29	.001	0.38	-0.06-	0.81	.088
Self-reported offending 15–18	0.22	0.11-	0.32	.001	0.36	0.23-	0.48	.001
Parental conviction					-0.41	-0.75-	-0.07	.019
Parental conviction * offspring conviction					2.58	1.60-	3.55	.001

### Interaction between labeling and convicted parent

**Fig 1:**
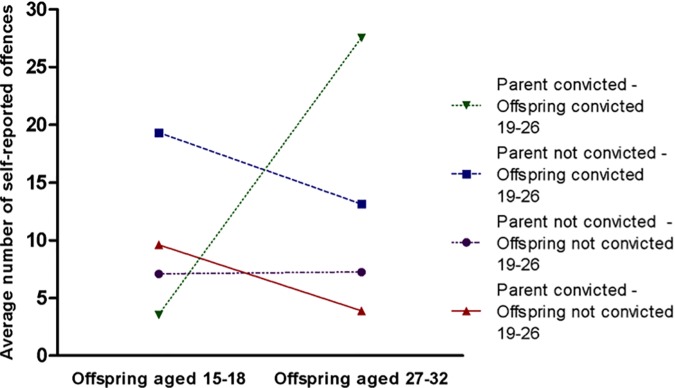
Interaction effect of parental conviction and offspring conviction on self-reported offending.

Model 2 in Table 1 demonstrates the result of the negative binomial regression analysis where the interaction between having a convicted parent and a conviction on subsequent self-reported offending was added. There was a strong interaction effect of a convicted parent and an offspring conviction on self-reported offending. Furthermore, the impact of a conviction at time 2 became an insignificant predictor when the interaction with a convicted parent was taken into account. When we ran separate analyses for the two groups to examine the impact of a conviction, a strong impact of a conviction on someone’s offending behavior was visible for the group whose parents had been convicted (*B* = 2.04, 95% CI = 1.13–2.95, *p* = .001), whereas there was no significant impact of a conviction for the group whose parents had not been convicted (*B* = -0.20, 95% CI = -0.73–0.34, *p* = .473). This interaction effect is also visible in Fig 1 and Table 2, which gives the average number of self-reported offences at the two ages for each of the four groups. Traditionally, when portraying an interaction effect, one would only report the outcome (self-reported offending between ages 27–32) for the four groups. However, since the outcome is heavily influenced by the previous level of self-reported offending, it is more appropriate to show the difference between the current and previous level of offending. The number of self-reported offences decreased between time 1 and time 3 for the first three groups, but the group who had a convicted parent and has been convicted at time 2 shows a sharp increase in self-reported offending between time 1 and time 3. Apparently, there was no labeling effect for the group whose parents have not been convicted, while there was a strong effect for children whose parents have been convicted. The results support hypothesis 2 and similarly show that hypothesis 1 is only supported for the group whose parents have been convicted and not for people whose parents have not been convicted.

**Table 2:** Interaction effect of parental conviction and offspring conviction on self-reported offending.

	Parent not convicted		Parent convicted	
	Offspring not convicted 19–26 (time 2)	Offspring convicted 19–26 (time 2)	Offspring not convicted 19–26 (time 2)	Offspring convicted 19–26 (time 2)
N	196	22	43	9
Mean number of self-reported offenses:				
Offspring aged 15–18 (time 1)	7.08 (18.53)	19.32 (31.09)	9.60 (17.25)	3.56 (6.48)
Offspring aged 27–32 (time 3)	7.25 (21.87)	13.14 (44.36)	3.91 (16.33)	27.56 (43.04)

We next added the control variables to the regression analysis. Table 3 displays the regression models, where each of the control variables was added separately. Table 4 displays the model including all variables. In each of the models, the interaction between a convicted parent and an offspring conviction remained significant. Indicators for serious offending for both son and parent as well as the combined parenting variable are significant predictors of self-reported offending between ages 27 and 32; son’s impulsiveness and family socioeconomic status are not. We see the same pattern when all the predictors are combined in one big model.

**Table 3:** Regression models adjusting for serious offending, son’s impulsive behavior, and socioeconomic status.

Dependent variable: SRO 27–32	B	95% CI B		*p*	B	95% CI B		*p*	B	95% CI B		*p*	B	95% CI B		*p*
Convicted 19–26	0.35	-0.08-	0.79	.111	0.36	-0.07-	0.80	.102	0.35	-0.81-	0.79	.111	0.36	-0.08-	0.79	.107
SRO 15–18	0.10	-0.11-	0.31	.358	0.36	0.24-	0.49	.001	0.67	0.24-	0.50	.001	0.35	0.22-	0.47	.001
Parental conviction	-0.50	-0.85-	-0.15	.005	-0.21	-0.57-	0.16	.260	-0.42	-0.76-	-0.07	.017	-0.35	-0.71-	0.01	.058
Parental conviction * offspring conviction	2.72	1.74-	3.71	.001	2.56	1.57-	3.54	.001	2.57	1.60-	3.54	.001	2.72	1.70-	3.74	.001
Offspring serious SRO	0.34	0.10-	0.58	.005												
Parent serious conviction					-1.11	-1.68-	-0.55	.001								
Offspring impulsiveness									0.39	-0.09-	0.87	.108				
Socioeconomic status													-0.34	-1.09-	0.41	.373

*Note* SRO: Self-reported offending

**Table 4:** Regression models adjusting for parenting risk factors and all predictors together.

Dependent variable: SRO 27–32	B	95% CI B		*p*	B	95% CI B		*p*
Convicted 19–26	-0.34	-0.80-	0.11	.138	-0.33	-0.79-	0.14	.168
SRO 15–18	0.27	0.15-	0.39	.001	0.03	-0.19-	0.24	.812
Parental conviction	-0.27	-0.61-	0.08	.130	-0.19	-0.58-	0.20	.348
Parental conviction * offspring conviction	3.27	2.31-	4.24	.001	3.17	2.16-	4.18	.001
Offspring serious SRO					0.33	0.09-	0.57	.006
Parent serious conviction					-1.16	-1.72-	-0.60	.001
Offspring impulsiveness					0.24	-0.27-	0.74	.359
Socioeconomic status					0.31	-0.50-	1.11	.453
Parenting	-0.29	-0.65-	-0.06	.105	-0.37	-0.75-	-0.01	.056

*Note* SRO: Self-reported offending.

## Discussion

This paper investigated the interaction between labeling and intergenerational transmission. In other words, we investigated the impact of a conviction on subsequent offending behavior and the interaction of a conviction and a convicted parent on subsequent offending. The results show that a conviction subsequently increased an individual’s self-reported offending behavior for the group of people whose parents had been convicted, but not for people whose parents had not been convicted. It is surprising that the significant impact of a conviction on someone’s subsequent offending behavior was only found for the people whose parents had been convicted, but not for the people whose parents had not been convicted. It appears that labeling theory only applies to people who are already disadvantaged by a convicted parent. There is a cumulative effect of having a convicted parent and being convicted yourself. As Bernburg and Krohn [17] emphasized: “structural location, such as race or social class, may provide people with differential means to resist deviant labeling in the face of official intervention.” A conviction does not automatically lead to deviant labeling, but also depends on other factors. When people are in a disadvantaged position “deficits and disadvantages pile up faster” [28]. The current study demonstrates strong support for this idea of differential labeling effects, supports the previous findings of Hagan and Palloni [46] and demonstrates that these differential labeling effects are present when one measures offspring offending up to age 32.

How can we explain this interaction between labeling and a convicted parent? Intergenerational transmission of criminal behavior can be explained by a combination of different mechanisms: potential biogenetic risk, social learning (or imitation of the parents’ behavior), official bias, and a criminogenic environment with risk factors for crime. Undoubtedly, intergenerational transmission stems from complex, reciprocal, and transactional forces spanning these different theoretical perspectives, leading to cumulative disadvantage for those children with criminal parents. If children also experience labeling because of a conviction, this basically adds additional burden to already disadvantaged individuals. These children seem to be more susceptible to labeling effects. If, in contrast, someone without such cumulative disadvantage coming from growing up with a convicted parent, experiences a criminal conviction, s/he may have more resources and therefore be more resilient to such an experience.

### Limitations and future directions

One assumption throughout the paper is the reliability of self-reported offending as a valid measure of someone’s actual offending behavior. Self-reports of criminal offending face challenges such as concealing, exaggerating or simply forgetting offending behavior [66], which are particularly problematic with long-term retrospective self-reports [67,68]. Furthermore, attributes of the respondent and of the crime might influence the willingness to admit, forget, and exaggerate offences. For example, people are less inclined to report sexual and fraud offences and more likely to exaggerate violent offences. This phenomenon could explain discrepancies between and official records and self-reports. Also, individuals who feel they have much to lose might be more inclined to present a pro-social image compared with offspring of convicted parents, who might feel stigmatized and labeled and will not hold back on self-reporting criminal behavior [66]. This scenario, however, would predict a smaller discrepancy between self-reported offending and official records for offspring of convicted parents, which was not found in the *CSDD* (see also [16]). It is important to realize that we might never know the true extent of offending behavior, even though numerous studies have shown that validity is high for prospective self-reports of white males as investigated in this sample. Nonetheless, self-reports are being widely used in criminological research and in this study are perceived as the nearest approximations of the respondents’ true offending behavior [68].

Furthermore, one could say that the increase found in self-reported offending after a conviction in the preceding period could be caused by an increased willingness to report offences rather than an increase in someone’s offending behavior. However, the respondents did not know that the researchers checked their criminal histories, therefore it seems unlikely that this knowledge could have influenced them. More importantly, previous analyses with the *CSDD* showed that, in general, the first self-report of an offence preceded the first conviction for it [61]. This implies that it is unlikely that the relationship found between a conviction and a subsequent increase in self-reported offending could be attributed to the tendency for convictions to make people more willing to admit offences in self-reports.

An important limitation of this study is the low number of people involved in the analyses to investigate labeling. The group of offspring with a conviction and a convicted parent consists of only nine people. Hitherto the *CSDD* is the only study used to examine the topic of labeling in combination with a convicted parent. This highlights the need to replicate these analyses with large longitudinal data sets over multiple generations.

Furthermore, it is important to realize that the results from this study might not be easily generalizable to today’s situation or to other countries. This sample of men was born in London around 1953 and their offending behavior was measured until age 32 (roughly 1986). They were mostly British and white and growing up in a specific society and time period. Family structures, communities and police and justice organizations have changed. Moreover, sentencing policies are different in current times and in other countries. One thing that we could not study using the *CSDD* is the effect of race or ethnicity. For example, Black or Hispanic offenders are more likely to be arrested, convicted, and imprisoned [69–78]. Similarly, people from a low socio-economic background seem to be arrested disproportionately often [77,78]. It would be advantageous to replicate this study using data from different periods and different countries to examine whether similar effects are visible. However, to be able to study labeling as well as intergenerational transmission of criminal behavior, a longitudinal study with information on offending for both generations is necessary, including self-reported offending for the offspring generation. Such studies are rare and the *CSDD* is one of the few studies that have collected such data. Related to this, it would be interesting to investigate this interaction between labeling and intergenerational transmission in samples using different age ranges. In this study, we used self-reported offending from ages 15–18 and 27–32, while looking at official convictions from ages 19–26. It would be interesting to see whether labeling effects might be stronger when people are convicted during their adolescent years, as we discussed in the introduction.

Similarly, unfortunately it was not possible investigate women. The *CSDD* does not have self-reports for sisters of the original 411 men. It would be desirable to replicate the current study using data on women to investigate labeling and intergenerational transmission for daughters. Women are less likely to be arrested than men, and women are at an advantage in several stages of delinquency case processing in the court [77,79–81]. It would be interesting to examine whether women also report a similar increase in self-reported offending after being convicted. Women who commit crime are less common, and because of this, women who do commit crime might be stigmatized more, based on the potential label as being disturbed rather than criminal, which could lead to an even stronger increase in self-reported behavior [82].

## Conclusion

Notwithstanding these limitations, this study is the first one in 25 years that investigated labeling and intergenerational transmission, showing this strong pattern of cumulative disadvantage of labeling and intergenerational transmission. What do these results mean to broader society? This study showed an increase in individuals’ offending behavior after labeling, and in particular for children of convicted parents. Although the aim of criminal justice agencies should be to decrease or prevent crime, by their actions the official agencies appear to increase offending behavior. These findings are particularly relevant when we consider official bias in intergenerational transmission, where offspring of convicted parents are at a higher risk of being targeted by the criminal justice system [16]. In their research in Edinburgh McAra & McVie [83–86] also discovered that the police appeared to unfairly target certain categories of young people, the “usual suspects”, by which they “serve to sustain and reproduce the very problems which the institution ostensibly attempts to contain or eradicate”.

Given that prison population numbers are at unprecedented levels and given that the number of convictions is still increasing in many nations, the effects of parental conviction on families and children are crucial societal concerns. This paper demonstrated that labeling might be a pivotal experience in the intergenerational transmission of criminal behavior. Instead of advancing this cycle of intergenerational crime by convicting these children disproportionally often, it is preferable to try to prevent the development of this behavior. Instead, interventions targeted at children of convicted parents would be a viable starting point. A first suggestion would be to provide family-based intervention programs, such as parent education and parent management training. Many scholars have shown the developmental merits of prevention programs [87–90]. Moreover, several reviews have shown that the monetary benefits of developmental prevention programs outweigh their monetary costs [89,91–97]. Monetary benefits can be wide-ranging from crime reduction to education (e.g. high school completion, college or university enrollment), employment (e.g. increased wages, tax revenue), and health (e.g. decreased use of public health care). If special consideration has to be given to children of convicted parents, it is preferable that this is positive and focused on preventing the offending behavior.
